# Morphine and alternative opioids in cancer pain: the EAPC recommendations: Expert Working Group of the Research Network of the European Association for Palliative Care

**DOI:** 10.1054/bjoc.2001.1680

**Published:** 2001-03

**Authors:** G W Hanks, F de Conno, N Cherny, M Hanna, E Kalso, H J McQuay, S Mercadante, J Meynadier, P Poulain, C Ripamonti, L Radbruch, J Roca i Casas, J Sawe, R G Twycross, V Ventafridda

**Affiliations:** Palliative Medicine, University of Bristol, Bristol Haematology and Oncology Centre, UK; Division of Rehabilitation, Pain Therapy and Palliative Care, Istituto Nazionale dei Tumori, Milano, Italy; Cancer Pain and Palliative Medicine Service, Shaare Zedek Medical Centre,Jerusalem, Israel; Pain Relief Research Unit, Kings College School of Medicine & Dentistry, University of London, UK; Pain Clinic, Helsinki University Hospital, Finland; University of Oxford, UK; Anaesthesia and Intensive Care Unit, Pain Relief and Palliative Care Unit, La Maddalena Cancer Centre and Home Care Programme, Societa per L'Assistenza al Malato Oncologico Terminale, Palermo, Italy; Department of Anaesthesiology, Intensive Care and Pain Treatment, Centre Oscar Lambret, Lille, France; Centre de diagnostic et de traitement de la douleur de l'adulte et de l'enfant, Institut Gustave-Roussy, Villejuif, France; Pain Clinic, Klinik für Anästhesiologie, Universität zu Koln, Germany; Hospital and Palliative Care Unit, Hospital de la Santa Creu, Barcelona,Spain; Huddinge University Hospital, Sweden; Sir Michael Sobell House, University of Oxford, UK; Floriani Foundation,Milano, Italy and the Steering Committee of the Research Network of the EAPC

**Keywords:** morphine, alternative opioids, European guidelines

## Abstract

An expert working group of the European Association for Palliative Care has revised and updated its guidelines on the use of morphine in the management of cancer pain. The revised recommendations presented here give guidance on the use of morphine and the alternative strong opioid analgesics which have been introduced in many parts of the world in recent years. Practical strategies for dealing with difficult situations are described presenting a consensus view where supporting evidence is lacking. The strength of the evidence on which each recommendation is based is indicated. © 2001 Cancer Research Campaign


*British Journal of Cancer* (2001) **84**(5), 587–593
© 2001 Cancer Research Campaign
doi: 10.1054/ bjoc.2001.1680, available online at http://www.idealibrary.com on IDEAL

http://www.bjcancer.com

### Morphine and alternative opioids in cancer pain: the EAPC recommendations

### Expert Working Group of the Research Network of the European Association for Palliative Care

**GW Hanks[1], F de Conno[2], N Cherny[3], M Hanna[4], E Kalso[5], HJ McQuay[6], S Mercadante[7], J Meynadier[8], P Poulain[9], C Ripamonti[2], L Radbruch[10], J Roca i Casas[11], J Sawe[12] RG Twycross[13] and V Ventafridda[14]**

[1]Palliative Medicine, University of Bristol, Bristol Haematology and Oncology Centre, UK; [2]Division of Rehabilitation, Pain Therapy and Palliative Care, Istituto Nazionale dei Tumori, Milano, Italy; [3]Cancer Pain and Palliative Medicine Service, Shaare Zedek Medical Centre, Jerusalem, Israel; [4]Pain Relief Research Unit, Kings College School of Medicine & Dentistry, University of London, UK; [5]Pain Clinic, Helsinki University Hospital, Finland; [6]University of Oxford, UK; [7]Anaesthesia and Intensive Care Unit, Pain Relief and Palliative Care Unit, La Maddalena Cancer Centre and Home Care Programme, Societa per L'Assistenza al Malato Oncologico Terminale, Palermo, Italy; [8]Department of Anaesthesiology, Intensive Care and Pain Treatment, Centre Oscar Lambret, Lille, France; [9]Centre de diagnostic et de traitement de la douleur de l'adulte et de l'enfant, Institut Gustave-Roussy, Villejuif, France; [10]Pain Clinic, Klinik für Anästhesiologie, Universität zu Koln, Germany; [11]Hospital and Palliative Care Unit, Hospital de la Santa Creu, Barcelona, Spain; [12]Huddinge University Hospital, Sweden; [13]Sir Michael Sobell House, University of Oxford, UK; [14]Floriani Foundation, Milano, Italy and the Steering Committee of the Research Network of the EAPC*

**Summary** An expert working group of the European Association for Palliative Care has revised and updated its guidelines on the use of morphine in the management of cancer pain. The revised recommendations presented here give guidance on the use of morphine and the alternative strong opioid analgesics which have been introduced in many parts of the world in recent years. Practical strategies for dealing with difficult situations are described presenting a consensus view where supporting evidence is lacking. The strength of the evidence on which each recommendation is based is indicated. © 2001 Cancer Research Campaign http://www.bjcancer.com

**Keywords**: morphine; alternative opioids; European guidelines

Most pain in cancer responds to pharmacological management using orally administered analgesics and adjuvants. Current treatment is based on the World Health Organization (WHO)'s concept of an 'analgesic ladder' which involves a stepwise approach to the use of analgesic drugs and is essentially a framework of principles rather than a rigid protocol (WHO, 1996). This allows considerable flexibility in the choice of specific drugs and the WHO ladder should be regarded as but one part of a comprehensive strategy for managing cancer pain. Symptomatic drug treatment is used in an integrated way with disease-modifying therapy and non-drug measures.

The most important part of the WHO method, and the reason for its success, is the efficient use of oral opioids for moderate to severe pain. Morphine is the benchmark 'step 3' opioid and in 1996 we published guidelines for the use of this drug in cancer pain management (Expert Working Group of the EAPC, 1996). Since our earlier publication, a number of alternatives to morphine have become available though these are generally not new molecules but novel formulations of existing drugs. There are few randomized controlled trials (RCTs) involving head to head comparisons between different opioids and this may make it difficult to choose the most appropriate drug for specific situations.

In view of the paucity of RCT data the European Association for Palliative Care's Expert Working Group on Opioid Analgesics has revised its recommendations for the use of morphine in cancer pain and extended them to cover the use of alternative opioids (Table 1). The strength of the evidence supporting each recommendation is indicated (Table 2).

*Received 7 December 2000*
*Accepted 8 January 2001*

*Correspondence to:* GW Hanks

### 1. The opioid of first choice for moderate to severe cancer pain is morphine C

Morphine is the standard 'step 3' opioid analgesic against which others are measured and is the most widely available in a variety of oral formulations. Morphine appears to have no clinically relevant ceiling effect to analgesia: doses of oral morphine may vary 1000-fold or more to achieve the same end point of pain relief.

#### *Unfounded fears associated with morphine*

Morphine has long been feared by both the general public and physicians (Lasagna, 1965). Underlying the fear is the mistaken belief that the problems associated with abuse of opioids are inextricably linked to therapeutic use. Concerns about addiction, excessive sedation, and respiratory depression have resulted in widespread avoidance or under-dosing. Yet extensive, carefully documented clinical experience has shown that these fears are unfounded (McQuay, 1999). Regular doses of morphine may be indicated and safely instituted early in the course of a patient's illness and continued for many months. Patients treated with morphine whose pain ameliorates can reduce the dose and discontinue it without difficulty.

*F De Conno (chair), A Caraceni, N Cherny, J Ferraz Goncalves, CJ Fürst, GW Hanks, S Kaasa, S Mercadante, JM Nunez Olarte, P Poulain, L Radbruch, C Ripamonti, F Stiefel.

**Table 1** Morphine and alternative opioids in cancer pain

1. The opioid of first choice for moderate to severe cancer pain is morphine. **C**
2. The optimal route of administration of morphine is by mouth. Ideally, two types of formulation are required: normal release (for dose titration) and modified release (for maintenance treatment). **C**
3. The simplest method of dose titration is with a dose of normal release morphine given every 4 hours and the same dose for breakthrough pain. This 'rescue' dose may be given as often as required (up to hourly) and the total daily dose of morphine should be reviewed daily. The regular dose can then be adjusted to take into account the total amount of rescue morphine. **C**
4. If pain returns consistently before the next regular dose is due the regular dose should be increased. In general, normal release morphine does not need to be given more often than every 4 hours and modified release morphine more often than 12 or 24 hours (according to the intended duration of the formulation). Patients stabilized on regular oral morphine require continued access to a rescue dose to treat 'breakthrough' pain. **A**
5. Several countries do not have a normal release formulation of morphine, though such a formulation is necessary for optimal pain management. A different strategy is needed if treatment is started with modified release morphine. Changes to the regular dose should not be made more frequently than every 48 hours, which means that the dose titration phase will be prolonged. **C**
6. For patients receiving normal release morphine every 4 hours, a double dose at bedtime is a simple and effective way of avoiding being woken by pain. **C**
7. Several modified release formulations are available. There is no evidence that the 12-hourly formulations (tablets, capsules or liquids) are substantially different in their duration of effect and relative analgesic potency. The same is true for the 24-hour formulations though there is less evidence to draw on. **A**
8. If patients are unable to take morphine orally the preferred alternative route is subcutaneous. There is generally no indication for giving morphine intramuscularly for chronic cancer pain because subcutaneous administration is simpler and less painful. **C**
9. The average relative potency ratio of oral morphine to subcutaneous morphine is between 1:2 and 1:3 (i.e. 20–30 mg of morphine by mouth is equianalgesic to 10 mg by s.c. injection). **C**
10. In patients requiring continuous parenteral morphine, the preferred method of administration is by subcutaneous infusion. **C**
11. Intravenous infusion of morphine may be preferred in patients:
    a. who already have an in-dwelling intravenous line;
    b. with generalized oedema;
    c. who develop erythema, soreness or sterile abscesses with subcutaneous administration;
    d. with coagulation disorders;
    e. with poor peripheral circulation. **C**
12. The average relative potency ratio of oral to intravenous morphine is between 1:2 and 1:3. **A**
13. The buccal, sublingual and nebulized routes of administration of morphine are not recommended because at the present time there is no evidence of clinical advantage over the conventional routes. **B**
14. Oral transmucosal fentanyl citrate (OTFC) is an effective treatment for 'breakthrough pain' in patients stabilized on regular oral morphine or an alternative step 3 opioid. **A**
15. Successful pain management with opioids requires that adequate analgesia be achieved without excessive adverse effects. By these criteria the application of the WHO and the EAPC guidelines (using morphine as the preferred step 3 opioid) permit effective control of chronic cancer pain in the majority of patients. In a small minority of patients adequate relief without excessive adverse effects may depend on the use of alternative opioids, spinal administration of analgesics or non-drug methods of pain control. **B**
16. A small proportion of patients develop intolerable adverse effects with oral morphine (in conjunction with a non-opioid and adjuvant analgesic as appropriate) before achieving adequate pain relief. In such patients a change to an alternative opioid or a change in the route of administration should be considered. **B**
17. Hydromorphone or oxycodone, if available in both normal release and modified release formulations for oral administration, are effective alternatives to oral morphine. **A**
18. Methadone is an effective alternative but may be more complicated to use compared with other opioids because of pronounced interindividual differences in its plasma half-life, relative analgesic potency and duration of action. Its use by non-specialist practitioners is not recommended. **C**
19. Transdermal fentanyl is an effective alternative to oral morphine but is best reserved for patients whose opioid requirements are stable. It may have particular advantages for such patients if they are unable to take oral morphine, as an alternative to subcutaneous infusion. **B**
20. Spinal (epidural or intrathecal) administration of opioid analgesics in combination with local anaesthetics or clonidine should be considered in patients who derive inadequate analgesia or suffer intolerable adverse effects despite the optimal use of systemic opioids and non-opioids. **B**

Daytime drowsiness, dizziness or mental clouding commonly occur at the start of treatment but resolve when patients are stabilized (usually within a few days). For most patients receiving stable doses of morphine effects on cognitive and psychomotor function are minimal. In particular, there are data indicating that patients' driving ability is not significantly impaired, in alert patients receiving a stable dose (Vainio et al, 1995). Similarly, nausea and vomiting, which occur in up to two-thirds of patients when morphine is started, usually resolve. The main continuing adverse effect from morphine is constipation, and the prophylactic use of a laxative is almost always required.

*Morphine: limitations*

The systemic availability of morphine by the oral route is poor (20–30%) and this contributes to a sometimes unpredictable onset of action and great interindividual variability in dose requirements and response (Glare and Walsh, 1991). Active metabolites may contribute to toxicity, particularly in patients with renal impairment (McQuay and Moore, 1997). And some types of pain do not always respond well or completely to morphine, notably neuropathic pain. However, none of the alternatives to morphine has so far demonstrated advantages which would make it preferable as the first line oral opioid for cancer pain. Morphine remains our first choice but for reasons of familiarity, availability and cost rather than proven superiority.

### 2. The optimal route of administration of morphine is by mouth. Ideally, two types of formulation are required: normal release (for dose titration) and modified release (for maintenance treatment) C

The oral route is the simplest and most acceptable to patients. There is large interindividual variation in kinetics (Säwe, 1986) and dynamics in cancer patients whose pain will also vary in

© 2001 Cancer Research Campaign

**Table 2** Strength and consistency of evidence supporting grades for each recommendation (as used by the Agency for Healthcare Policy and Research, USA)

| | |
|---|---|
| A: | requires at least one randomized controlled trial as part of a body of literature of overall good quality and consistency addressing the specific recommendation (evidence levels Ia and Ib). |
| B: | requires the availability of well-conducted clinical studies but no randomised clinical trials on the topic of recommendation (evidence levels IIa, IIb and III). |
| C: | requires evidence obtained from expert committee reports or opinions and/or clinical experiences of respected authorities. Indicates an absence of directly applicable clinical studies of good quality (evidence level IV). |
| Category of evidence | |
| Ia | evidence from meta-analysis of randomised controlled trials. |
| Ib | evidence from at least one randomised controlled trial. |
| IIa | evidence from at least one controlled study without randomization. |
| IIb | evidence from at least one other type of quasi-experimental study. |
| III | evidence from non-experimental descriptive studies, such as comparative studies, correlation studies, and case-control studies. |
| IV | evidence from expert committee reports or opinions or clinical experience of respected authorities, or both. |

severity so that the dose must be titrated against effect for each patient, and the starting dose will be determined by previous analgesic treatment. Patients changing from regular administration of a step 2 opioid (in combination with a non-opioid) will usually start with 10 mg every 4 hours. If step 2 of the analgesic ladder is omitted 5 mg every 4 hours may suffice, whereas patients converted from another step 3 opioid will require more.

During dose titration it is preferable to use a formulation of morphine that has a rapid onset and a short duration of action to allow steady state to be achieved as quickly as possible. Normal release formulations fulfil these requirements. Peak plasma concentrations usually occur within the first hour after oral administration (Hoskin et al, 1989), with a reasonably rapid onset of analgesia which then lasts for about 4 hours. In contrast modified release morphine formulations produce a delayed peak plasma concentration after 2–6 hours (Hoskin et al, 1989; Gourlay et al, 1997), the peak is attenuated (Hoskin et al, 1989), and analgesia lasts for 12 or 24 hours (Hanks, 1990; Gourlay et al, 1997). This means that with modified release morphine it is more difficult to rapidly assess the adequacy of analgesia and to adjust the dose during the dose-finding period.

### 3. The simplest method of dose titration is with a dose of normal release morphine given every 4 hours and the same dose for breakthrough pain. This 'rescue' dose may be given as often as required (up to hourly) and the total daily dose of morphine should be reviewed daily. The regular dose can then be adjusted to take into account the total amount of rescue morphine C

The plasma elimination half-life of morphine is 2–4 hours (Glare and Walsh, 1991) and steady state is achieved within 4–5 half-lives (that is within 24 hours) (Säwe et al, 1983) after the start of treatment and following dose adjustment. This is an important interval in which to re-evaluate a patient and adjust the daily dose. This method of dose titration avoids the need to remember predetermined increments and has been shown to be safe and effective.

During the dose titration phase using 4-hourly normal release morphine, the full 4-hourly dose should be used for 'rescue'. The frequency with which the rescue dose can be offered depends on the route of administration and the time to peak effect. Oral rescue doses are usually offered up to every 1–2 hours and parenteral doses (equivalent to the 4-hourly parenteral dose) can be offered as frequently as every 15–30 minutes.

### 4. If pain returns consistently before the next regular dose is due the regular dose should be increased. In general, normal release morphine does not need to be given more often than every 4 hours and modified release morphine more often than 12 or 24 hours (according to the intended duration of the formulation). Patients stabilized on regular oral morphine require continued access to a rescue dose to treat 'breakthrough' pain A

The drug regimen should be as simple as possible. Increasing the frequency of administration may adversely affect compliance and convenience for the patient. Increasing the dose allows a 4-hourly or 12- or 24-hourly regimen to be achieved without producing troublesome adverse effects associated with the increase in peak blood concentrations (Hanks, 1990; Gourlay et al, 1997). A few patients receiving 12-hourly formulations do not seem to achieve a 12 hour duration of analgesia and require administration every 8 hours. Occasionally patients taking a high dose prefer dosing every 8 hours to avoid taking too many tablets at a time, particularly in countries where no high-dose formulations are available.

Patients receiving regular oral opioids may experience acute episodic breakthrough pain which may be a function of the pain itself or may be precipitated by some voluntary act such as weight-bearing or movement. There are no RCT data to establish the appropriate dose of morphine for breakthrough pain and anecdotal experience supports the use of doses varying from 30 to 100% of the 4-hourly dose (Portenoy and Hagan, 1990). It may be that the optimal dose for breakthrough pain can only be determined by titration but we suggest that a simple approach is to use the equivalent 4-hourly dose of morphine (as during the dose-finding period).

*© 2001 Cancer Research Campaign*

**5. Several countries do not have a normal release formulation of morphine, though such a formulation is necessary for optimal pain management. A different strategy is needed if treatment is started with modified release morphine. Changes to the regular dose should not be made more frequently than every 48 hours, which means that the dose titration phase will be prolonged C**

Total daily dose requirements should be estimated on the basis of previous analgesic intake. Breakthrough pain is managed with single doses of a non-opioid (non-steroidal anti-inflammatory drug or paracetamol) as required, or with another short-lasting strong opioid available for oral administration (such as oxycodone), or with oral or rectal administration of morphine injection solution (or a solution of morphine made from powder, if this is available and cheaper).

**6. For patients receiving normal release morphine every 4 hours, a double dose at bedtime is a simple and effective way of avoiding being woken by pain C**

No formal investigations of this practice are available. However, it has been widely adopted (Twycross, 1984) and does not seem to cause problems (Regnard and Badger, 1987).

**7. Several modified release formulations are available. There is no evidence that the 12-hourly formulations (tablets, capsules or liquids) are substantially different in their duration of effect and relative analgesic potency. The same is true for the 24-hour formulations though there is less evidence to draw on A**

Although in principle it is unwise to change between preparations when using modified release products because of possible variations in release profiles and oral bioavailability there is no consistent evidence that the various oral formulations of morphine designed for administration every 12 hours have a different pharmacokinetic or pharmacodynamic profile in patients (Collins et al, 1998).

Several once-a-day formulations of morphine have also been developed. There are significant differences between some in their pharmacokinetic profiles (Gourlay et al, 1997) but there is no evidence that this is reflected in clinically significant differences in patients: they appear to be equivalent in efficacy and in the duration of effect.

**8. If patients are unable to take morphine orally the preferred alternative route is subcutaneous. There is generally no indication for giving morphine intramuscularly for chronic cancer pain because subcutaneous administration is simpler and less painful C**

The advantages of subcutaneous injection are that a smaller needle is required, the chance of damage to nerves is less so that the site of injection is not crucial, and the possibility of inadvertent intravenous injection is less because veins can be seen more easily. Absorption is similar and peak plasma concentrations are achieved within 15–30 minutes, with a more rapid onset of drug action than after oral administration.

Alternative drugs, particularly diamorphine (Twycross, 1994) (in the UK) and hydromorphone (Moulin et al, 1991), may be preferred for parenteral administration because they are more soluble than morphine so that a smaller volume injection is necessary. Transdermal fentanyl may be a useful non-invasive alternative in patients with stable opioid requirements.

Rectal administration may be preferred by some patients. The bioavailability of morphine and duration of effect is similar to the oral route and the equianalgesic dose by oral and rectal routes is the same (Ripamonti and Bruera, 1991).

**9. The average relative potency ratio of oral morphine to subcutaneous morphine is between 1:2 and 1:3 (i.e. 20–30 mg of morphine by mouth is equianalgesic to 10 mg by s.c. injection) C**

Drugs administered by parenteral routes do not undergo pre-systemic (‘first pass’) metabolism. The relative potency ratio of oral to parenteral morphine has been highly controversial (Hanks et al, 1987; Kaiko, 1988; Twycross, 1988). It seems that relative potency varies according to the circumstances in which morphine is used and between individual patients. When converting from oral morphine to subcutaneous morphine, the dose should be divided by three to get a roughly equianalgesic effect, but upward or downward adjustment of the dose may then be required.

**10. In patients requiring continuous parenteral morphine, the preferred method of administration is by subcutaneous infusion C**

Portable battery-operated syringe drivers are now widely used to administer drugs by continuous slow infusion to patients with advanced cancer who are unable to take oral medication (Dover, 1987).

**11. Intravenous infusion of morphine may be preferred in patients: a. who already have an in-dwelling intravenous line; b. with generalized oedema; c. who develop erythema, soreness or sterile abscesses with subcutaneous administration; d. with coagulation disorders; e. with poor peripheral circulation C**

Subcutaneous infusions have several advantages over intravenous infusions: venous access is not required, close supervision is unnecessary, and infection is unlikely. However, intravenous infusion may have advantages in the specific circumstances listed above.

Transdermal fentanyl may be a useful non-invasive alternative in patients with stable opioid requirements.

**12. The average relative potency ratio of oral to intravenous morphine is between 1:2 and 1:3 A**

The relative potency by intravenous and subcutaneous routes is the same. When converting from oral to intravenous morphine the oral dose should be divided by three (Kalso and Vainio, 1990).

 © 2001 Cancer Research Campaign

**13. The buccal, sublingual and nebulized routes of administration of morphine are not recommended because at the present time there is no evidence of clinical advantage over the conventional routes B**

The absorption of morphine by these routes is unpredictable (Chrubasik et al, 1988; Ripamonti and Bruera, 1991), and they are best avoided for this drug. In contrast, the highly lipophilic drugs methadone, fentanyl and buprenorphine are well absorbed sublingually and buprenorphine is used by this route. Sublingual buprenorphine may be a useful alternative to low-dose oral morphine for patients who have difficulty swallowing, but experience of long-term use in cancer pain is limited.

**14. Oral transmucosal fentanyl citrate (OTFC) is an effective treatment for 'breakthrough pain' in patients stabilized on regular oral morphine or an alternative step 3 opioid A**

OTFC produces a rapid onset of analgesia in 5–15 minutes with a short duration of action of about 2 hours. This is a new treatment with which there is very limited clinical experience but good RCT data to support efficacy (Portenoy et al, 1991). More safety data are required from wider and longer term clinical use.

**15. Successful pain management with opioids requires that adequate analgesia be achieved without excessive adverse effects. By these criteria the application of the WHO and the EAPC guidelines (using morphine as the preferred step 3 opioid) permit effective control of chronic cancer pain in the majority of patients. In a small minority of patients adequate relief without excessive adverse effects may depend on the use of alternative opioids, spinal administration of analgesics or non-drug methods of pain control B**

A number of observational studies have been carried out to validate the WHO approach and have involved some 8000 patients in different countries and different clinical environments (Jadad and Browman, 1995; Mercadante, 1999). Reported response rates (for adequate analgesia) have varied between 71 and 100%

**16. A small proportion of patients develop intolerable adverse effects with oral morphine (in conjunction with a non-opioid and adjuvant analgesic as appropriate) before achieving adequate pain relief. In such patients a change to an alternative opioid or a change in the route of administration should be considered B**

In some patients experiencing troublesome adverse effects a reduction in dose of morphine may alleviate these effects while maintaining adequate analgesia (Hanks, 1991). If this is unsuccessful switching to an alternative opioid agonist may allow titration to adequate analgesia without the same disabling effects.

Dose-limiting adverse effects most often involve CNS toxicity (drowsiness, cognitive impairment, confusion, hallucinations, myoclonic jerks). In some centres it has been found necessary or beneficial to change to an alternative opioid in up to 40% of patients (de Stoutz et al, 1995). Sometimes several changes of drug are employed and the term 'opioid rotation' has been coined to describe this practice. Others estimate that the proportion of patients who develop intolerable adverse effects with oral morphine is much smaller.

Switching between opioids complicates pain management and this is a disadvantage for non-specialists (for whom it is not recommended without expert advice). Appropriate strategies for the management of this situation are the subject of a separate expert report (Expert Working Group of the EAPC, in press).

**17. Hydromorphone or oxycodone, if available in both normal release and modified release formulations for oral administration, are effective alternatives to oral morphine A**

Hydromorphone is a semi-synthetic congener of morphine and a potent μ-selective agonist similar to morphine and between 5 and 10 times as potent (Houde, 1986). There appear to be no major differences between hydromorphone and morphine in terms of efficacy and adverse effects when used in equianalgesic doses.

Oxycodone is a semi-synthetic congener of morphine which until recently was most often prescribed in low-dose combination products (with a non-opioid) for oral administration or as a rectal suppository. In some countries it has been more widely used as a single agent to treat post-operative pain and cancer pain. It has now become available in new oral formulations (normal and modified release). Oxycodone is similar to morphine in terms of analgesia and adverse effects (Kalso and Vainio, 1990; Hanks and Hawkins, 2000). Because of its better systemic availability (about 60–90%) the equianalgesic dose of oral oxycodone is between half and two-thirds that of oral morphine (Bruera et al, 1998).

**18. Methadone is an effective alternative but may be more complicated to use compared with other opioids because of pronounced interindividual differences in its plasma half-life, relative analgesic potency and duration of action. Its use by non-specialist practitioners is not recommended C**

Methadone is a synthetic opioid widely available in oral formulations. It has no known active metabolites. There is a discrepancy between the duration of its initial analgesic effect (4–6 hours) and its plasma elimination half-life which averages approximately 24 hours with a range of 17 to over 100 hours (Plummer et al, 1988). The drug accumulates on chronic dosing so that it should not be given more frequently than 8-hourly (DeConno et al, 1996) to avoid potential adverse effects. When switching from another opioid it is often difficult to accurately determine the equianalgesic dose (Ripamonti et al, 1998), particularly in patients tolerant to high doses of opioids.

**19. Transdermal fentanyl is an effective alternative to oral morphine but is best reserved for patients whose opioid requirements are stable. It may have particular advantages for such patients if they are unable to take oral morphine, as an alternative to subcutaneous infusion B**

Fentanyl is a semi-synthetic opioid and an established intravenous anaesthetic and analgesic drug which is about 80 times as potent as

*© 2001 Cancer Research Campaign*

parenteral morphine. It is not used by mouth because it rapidly undergoes extensive first-pass metabolism. The low molecular weight and high lipid solubility of fentanyl facilitate absorption through the skin. After application fentanyl is undetectable in the systemic circulation for 1 to 2 hours, but then serum levels rise with analgesic effects evident within 8 to 16 hours and steady state is achieved at 72 hours (Lehmann and Zech, 1992). Each patch is applied for 3 days. An intradermal depot develops so that following removal of the patch serum levels take about 16 hours to drop to 50%.

Transdermal fentanyl is effective and well tolerated in the management of cancer pain, but is generally less flexible than shorter-acting preparations. Although the 3 day duration of action is an important advantage for patients with stable opioid requirements it can complicate management of patients with unstable pain whose opioid requirements are fluctuating. There is some experimental and clinical evidence that transdermal fentanyl is associated with less constipation than morphine (Megens et al, 1998).

### 20. Spinal (epidural or intrathecal) administration of opioid analgesics in combination with local anaesthetics or clonidine should be considered in patients who derive inadequate analgesia or suffer intolerable adverse effects despite the optimal use of systemic opioids and non-opioids B

Spinal opioids (± a local anaesthetic or clonidine) are indicated in patients who have intolerable adverse effects with systemically administered opioids. The addition (by the epidural route) of a local anaesthetic may be particularly useful in managing movement-related, incident pain (Mercadante, 1999b), and of clonidine for neuropathic pain (Eisenach et al, 1995).

### ACKNOWLEDGEMENT

We should like to thank Deborah Ashby for her considerable assistance in the production of this paper.

### REFERENCES

Bruera E, Belzile M, Pituskin E, Fainsinger R, Darke A, Harsanyi Z, Babul N, Ford I (1998) Randomised double blind crossover trial comparing safety and efficacy of oral controlled release oxycodone with controlled release morphine in patients with cancer pain. *J Clin Oncol* **16**: 3222–3229.

Chrubasik J, Wust H, Friedrich G and Geller E (1988) Absorption and bioavailability of nebulized morphine. *Br J Anaesth* **61**: 228–230

Collins SL, Faura CC, Moore A and McQuay HJ (1998) Peak plasma concentrations after oral morphine: a systematic review. *J Pain Symptom Manage* **16**: 388–402

De Conno F, Groff L, Brunelli C, Zecca E, Ventafridda V and Ripamonti C (1996) Clinical experience with oral methadone administration and the treatment of pain in 196 advanced cancer patients. *J Clin Oncol* **14**: 2836–2842

de Stoutz ND, Bruera E and Suarez-Almazor M (1995) Opioid rotation for toxicity reduction in terminal cancer patients. *J Pain Symptom Manage* **10**: 378–384

Dover SB (1987) Syringe driver in terminal care. *BMJ* **294**: 553–555

Eisenach JC, DuPen S, Dubois M, Miguel R and Allin D (1995) Epidural clonidine analgesia for intractable cancer pain. *Pain* **61**: 391–399

Expert Working Group of the European Association for Palliative Care (1996) *Morphine in cancer pain: modes of administration*. *BMJ* **312**: 823–826

Expert Working Group of the Research Network of the European Association for Palliative Care. Strategies to relieve the adverse effects of oral morphine. *J Clin Oncol*, in press

Glare PA and Walsh TD (1991) Clinical pharmacokinetics of morphine. *Ther Drug Monit* **13**: 1–23

Gourlay GK, Cherry D, Onley MM, Tordoff SG, Conn DA, Hood GM and Plummer JL (1997) Pharmacokinetics and pharmacodynamics of twenty-four-hourly Kapanol compared to twelve-hourly MS Contin in the treatment of severe cancer pain. *Pain* **69**: 295–302

Hanks GW (1990) Controlled release morphine tablets in chronic cancer pain: a review of controlled clinical trials. In: Benedetti C, Chapman CR, Giron G (eds). Opioid Analgesia. Recent Advances in Systemic Administration (Advances in Pain Research and Therapy 14) pp 269–274. New York: Raven Press

Hanks GW (1991) Opioid responsive and opioid-non-responsive pain in cancer. *Br Med Bull* **47**: 718–731

Hanks GW and Hawkins C (2000) Agreeing a gold standard in the management of cancer pain: the role of opioids. In: Hillier R, Finlay I, Welsh J, Miles A (eds). UK Key Advances in Clinical Practice Series 2000. *The Effective Management of Cancer Pain* pp 57–75. London: Aesculapius Medical Press

Hanks GW, Hoskin PJ, Aherne GW, Turner P and Poulain P (1987) Explanation for potency of oral morphine on repeated dosage? *Lancet* **ii**: 723–725

Hoskin PJ, Hanks GW, Aherne GW, Chapman D, Littleton P and Filshie J (1989) The bioavailability and pharmacokinetics of morphine after intravenous, oral and buccal administration in healthy volunteers. *Br J Clin Pharmacol* **27**: 499–505

Houde RW (1986) Clinical analgesic studies of hydromorphone. In: Foley KM & Inturrisi CE (eds). *Advances in pain research and therapy*. Vol 8 pp 129–135 New York: Raven Press

Jadad AR and Browman GP (1995) The WHO analgesic ladder for cancer pain management. Stepping up the quality of its evaluation. *JAMA* **274**: 1870–1873

Kaiko RF (1988) The therapeutic equivalence of IM and PO administration of morphine – 1:3 or 1:6. *J Palliat Care* **4**: 64–66

Kalso E and Vainio A (1990) Morphine and oxycodone hydrochloride in the management of cancer pain. *Clin Pharmacol Ther* **47**: 639–664

Lasagna L (1965) Addicting drugs and medical practice: towards the elaboration of realistic goals and the eradication of myths, mirages and half-truths. In: Wilner DM, Kassebaum GG (eds) *Narcotics*. pp 53–56 New York: McGraw

Lehmann KA and Zech D (1992) Transdermal fentanyl: clinical pharmacology. *J Pain Symptom Manage* **7**: S8–S16

McQuay H (1999) Opioids in pain management. *Lancet* **353**: 2229–2232

McQuay HJ and Moore RA (1997) Opioid problems, and morphine metabolism and excretion. In: Dickenson AH, Besson J-M (eds) *Handbook of Experimental Pharmacology*, **130** pp 335–360. Berlin: Springer-Verlag

Megens A, Artois K and Vermeire J et al (1998) Comparison of the analgesic and intestinal effects of fentanyl and morphine in rats. *J Pain Symptom Manage* **15**: 253–257

Mercadante S (1999a) Pain treatment and outcomes for patients with advanced cancer who receive follow up care at home. *Cancer* **85**: 1849–1858.

Mercadante S (1999b) Problems of long term spinal opioid treatment in advanced cancer patients. *Pain* **79**: 1–13

Moulin DE, Kreeft JH, Murray PN and Bouquillon AI (1991) Comparison of continuous subcutaneous and intravenous hydromorphone infusions for management of cancer pain. *Lancet* **337**: 465–468

Plummer JL, Gourlay GK, Cherry DA and Cousins MJ (1988) Estimation of methadone clearance: application in the management of cancer pain. *Pain* **33**: 313–322

Portenoy RK and Hagan NA (1990) Breakthrough pain: definition, prevalence and characteristics. *Pain* **41**: 273–281

Portenoy RK, Payne R, Coluzzi P et al (1999) Oral transmucosal fentanyl citrate (OTFC) for the treatment of breakthrough pain in cancer patients: a controlled dose titration study. *Pain* **79**: 303–312

Regnard CFB and Badger C (1987) Opioids, sleep and the time of death. *Palliat Med* **1**: 107–110

Ripamonti C and Bruera E (1991) Rectal, buccal and sublingual narcotics for the management of cancer pain. *J Palliat Care* **7**: 30–35

Ripamonti C, Groff L, Brunelli C, Polastri D, Stavrakis A and De Conno F (1998) Switching from morphine to oral methadone in treating cancer pain: what is the equianalgesic dose ratio? *J Clin Oncol* **16**: 3216–3221

Säwe J (1986) High dose morphine and methadone in cancer patients. Clinical pharmacokinetic considerations of oral treatment. (1986) *Clin Pharmacokin* **11**: 87–106

Säwe J, Dahlström B and Rane A (1983) Steady state kinetics and analgesic effect of oral morphine in cancer patients. *Eur J Clin Pharmacol* **24**: 537–542

Twycross RG (1984) Control of pain. *J R Coll Physicians Lond* **18**: 32–39

 © 2001 Cancer Research Campaign

Twycross RG (1988) The therapeutic equivalence of oral and subcutaneous/intramuscular morphine sulphate in cancer patients. *J Palliat Care* **4**: 67–68

Twycross R (1994) Pain relief in advanced cancer, pp 261–266: Edinburgh: Churchill Livingstone

Vainio A, Ollila J, Matikainen E, Rosenberg P and Kalso E (1995) Driving ability in cancer patients receiving long-term morphine analgesia. *Lancet* **346**: 667–670

World Health Organisation (1996) *Cancer Pain Relief*, 2nd edition. WHO: Geneva

*© 2001 Cancer Research Campaign*
